# Association of a Variant of *CNR1* Gene Encoding Cannabinoid Receptor 1 With Gilles de la Tourette Syndrome

**DOI:** 10.3389/fgene.2020.00125

**Published:** 2020-03-04

**Authors:** Natalia Szejko, Jakub Piotr Fichna, Krzysztof Safranow, Tomasz Dziuba, Cezary Żekanowski, Piotr Janik

**Affiliations:** ^1^ Department of Neurology, Medical University of Warsaw, Warsaw, Poland; ^2^ Department of Bioethics, Medical University of Warsaw, Warsaw, Poland; ^3^ Laboratory of Neurogenetics, Department of Neurodegenerative Disorders, Mossakowski Medical Research Centre, Polish Academy of Sciences, Warsaw, Poland; ^4^ Department of Biochemistry and Medical Chemistry, Pomeranian Medical University, Szczecin, Poland

**Keywords:** Gilles de la Tourette syndrome, endocannabinoids, association study, *CNR1* gene, CB1cannabinoid receptor 1

## Abstract

**Background:**

Gilles de la Tourette syndrome (GTS) is a neuropsychiatric disorder of unknown etiology, although a major role of genetic factors has been established. Cannabis-based medicines may alleviate GTS-associated tics and variants of CNR1 gene encoding central cannabinoid receptor (CB1) are believed to be a risk factor for the development of some neurodevelopmental diseases. Our aim was to test the association of selected *CNR1* gene variants with GTS.

**Material and Methods:**

The cohort of GTS cases comprised 262 unrelated patients aged 3–53 years (mean age: 18.3 ± 9.1 years; 204 males (77.9%), 126 (48.1%) adults defined as ≥18 years). As a control group we enrolled 279 unrelated, ethnically and gender matched individuals with no diagnosed mental, neurological or general disorder, aged 13–54 years (mean age: 22.5 ± 3.0 years; 200 males, (74.1%). Both study and control groups were selected from Polish population, which is ethnically homogenous subgroup of Caucasian population. Four single nucleotide polymorphisms (SNPs) in *CNR1* were selected: rs2023239, rs2180619, rs806379, and rs1049353 based on minor allele frequency in general population >15%. These variants were genotyped using a real-time quantitative polymerase chain reaction system (TaqMan SNP genotyping assay).

**Results:**

We found significant association of GTS clinical phenotype with rs2023239 variant. Minor allele C and CT+CC genotypes were found significantly more often in GTS patients compared to controls (17.4 vs 11.1%, p=0.003 and 32.8 vs 20.4%, p=0.001, respectively), and the difference remained significant after correction for multiple testing. C allele of rs2023239 polymorphism of the CNR1 gene was associated with the occurrence of tics. There were no statistically significant associations for rs806379, rs1049353 or rs2180619 variants.

**Conclusion:**

Our findings suggest that C allele of rs2023239 polymorphism of the *CNR1* gene is a risk factor of GTS in Polish population. The variant can be potentially associated with abnormal endocannabinoid transmission, which is suspected to be one of the causes of GTS.

## Introduction

Gilles de la Tourette syndrome (GTS) is neuropsychiatric disorder in which motor and vocal tics occur. They are usually accompanied by psychiatric comorbidities such as attention-deficit hyperactivity disorder (ADHD), obsessive–compulsive disorder (OCD), depression or self-injury. Segregation analysis in GTS-risk families suggests a complex, polygenic mode of inheritance involving various *loci* (Mcmahon et al., 1996). Susceptibility genes associated with GTS include: Slit and Trk-like1 (*SLITRK1*), L-histidine decarboxylase (*HDC*), mitochondrial inner membrane protease subunit 2 (*IMMP2L*), neuroligin 4, X-linked (*NLGN4X*), contactin-associated protein 2 (*CNTNAP2*) (for details consult review by Paschou et. al (Paschou et al., 2014), cadherin EGF LAG seven-pass G-type receptor 3 (*CELSR3*) (Willsey et al., 2017; Wang et al., 2018), WW and C2 domain containing 1 (*WWC1)*, Nipped-B-like (*NIPBL*), fibronectin 1 (*FN1*) (Willsey et al., 2017), arylacetamide deacetylase (*AADAC*) (Yuan et al., 2018) and others.

Abnormal dopaminergic neurotransmission in the cortex and in the basal ganglia interacting with other neuronal pathways, including endocannabinoid system (ECS), seems to be crucial in GTS pathophysiology (Martino and Leckman, 2013). Endocannabinoids mediate two pathways in the basal ganglia: neurons of the direct pathway expressing dynorphin and D1 dopamine receptor, as well as the indirect pathway neurons expressing enkephalin and D2 receptor (Steiner and Gerfen, 1998; Mechoulam and Parker, 2013). There are many further indications for an involvement of ECS in GTS (Sandyk, 1988; Leckman et al., 1988; Haber and Wolfer, 1992; Muller-Vahl, 1998; Muller-Vahl, 2002; Müller-Vahl et al., 2002; Hasan et al., 2010; Brunnauer et al., 2011; Mechoulam and Parker et al., 2013; Trainor et al., 2016; Jakubovski and Müller-Vahl, 2017; Kanaan et al., 2017). Some post-mortem studies support cannabinoid dysregulation in GTS, as decreased immunoreactivity with dynorphin A [1–17] has been detected in the striatal area of GTS patients (Haber and Wolfer, 1992). Furthermore, increased levels of dynorphin A [1–8] were found in the cerebrospinal fluid of GTS patients which correlated with OCD severity (Leckman et al., 1988). The ECS involvement in GTS is also indicated by a neuroimaging study using single photon emission computed tomography (SPECT) with [^123^I]AM281 (Berding et al., 2004). The clinical efficacy of cannabis-based medicines (CBM) also supports the involvement of ECS (Müller-Vahl et al., 2002; Müller-Vahl et al., 2003). Additionally, a number of retrospective reports on self-medication with CBM (Hemming and Yellowlees, 1993; Muller-Vahl, 2002; Brunnauer et al., 2011; Jakubovski and Müller-Vahl, 2017) as well as case reports (Muller-Vahl, 2002; Hasan et al., 2010; Brunnauer et al., 2011; Trainor et al., 2016; Jakubovski and Müller-Vahl, 2017; Kanaan et al., 2017) suggest effectiveness of CBM in GTS.

The two main cannabinoid receptors are CB1 and CB2, cannabinoid receptors type 1 and 2. CB1 receptor is mostly present in the central nervous system, with a high density in basal ganglia (Pertwee, 2006), the brain region implicated in movement control, while CB2 receptor is mainly present in the immune system and in hematopoietic cells (Russo, 2013) where it modulates cytokine release (Suárez-Pinilla et al., 2015). Variants of the *CNR1* gene encoding CB1 receptor have been associated with various psychiatric and neurological diseases (Gadzicki et al., 1999; Gadzicki et al., 2004; Domschke et al., 2008; Evans et al., 2016; Ruiz-Contreras et al., 2017; Yao et al., 2018) (see **Table 1**). Gadzicki et al. (1999) were the first to investigate polymorphisms in a coding exon of the *CNR1* gene finding the 1359(G/A) polymorphism in German GTS patients. Posteriorly they focused on *CNR1* variants in a larger group of GTS patients (Janik et al., 2014). However, they failed to find a correlation between those variants and GTS.

**Table 1:** SNPs selected for the study.

SNP	MAF (NFE)	Localization	Associated neuropsychiatric disorders
rs2023239	16%	intron	substance dependence (Haughey et al., 2008; Filbey et al., 2010), eating disorders (Müller et al., 2007), schizophrenia (Yu et al., 2013), impulsivity (Ehlers et al., 2007), depression (Icick et al., 2015), cyclic vomiting syndrome (Wasilewski et al., 2017)
rs2180619	40%	promoter	anxiety (Lazary et al., 2009), substance dependence (Chen et al., 2008), eating disorders (Müller et al., 2008), personality disorders (Yao et al., 2018)
rs806379	47%	intron	anxiety (Lester et al., 2017), substance dependence (Evans et al., 2016), schizophrenia (Yu et al., 2013), impulsivity (Buchmann et al., 2015)
rs1049353	27%	exon (synonymous)	substance dependence (Hindocha et al., 2019), eating disorders (Sadeghian et al., 2018), schizophrenia (Suárez-Pinilla et al., 2015), depression (Mitjans et al., 2013), multiple sclerosis (Varadé et al., 2012)

MAF, minor allele frequency, in gnomAD database; associated neuropsychiatric disorders based on SNPedia database.

SNP, single nucleotide polymorphism.

Despite that negative outcome we decided to revise the question of a possible role of CB1 receptor in GTS. We focused on the CB1 receptor as it is mainly expressed in the brain and mediates inhibition of dopaminergic, glutaminergic, and GABAergic neurotransmission in CNS (Haughey et al., 2008). We hypothesized that ECS dysregulation in GTS could be related to some minor variants of the *CNR1* gene and therefore tested four such variants as possible risk factors of GTS in Polish GTS patients.

## Material and Methods

### Ethics Statement

The study has been approved by the Ethics Committee of the Medical University of Warsaw (KB/2/2007, KB/53/A/2010, KB/63/A/2018) and has therefore been performed in accordance with the ethical standards laid down in the 1964 Declaration of Helsinki and its later amendments. All participants aged ≥18 years signed an informed consent form prior to their inclusion in the study, and legal guardians gave a written consent on behalf of individuals under 18.

### Study Participants

The study group comprised 262 unrelated GTS patients aged 3–53 years (mean age: 18.3 ± 9.1 years; 204 males (77.9%), 126 (48.1%) adults defined as ≥18 years). The family history of tics was positive in 142 (54.8%) patients, and was unknown for three patient. The family history of OCD or obsessive–compulsive behavior (OCB) was available from 125 patients, and was positive for 37 (29.6%) patients. The mean age of tic onset was 6.9 ± 3.0 years. Two hundred and three (77.8%) patients had at least one of the following co-morbidities: OCD/OCB n = 127 (48.7%), ADHD n=90 (34.5%), non-OCD anxiety disorder n = 84 (32.2%), learning disorder n=64 (24.5%), depression n = 37 (14.2%), conduct disorder n=16 (6.1%), while 58 (22.2%) had none of the above. Yale Global Tic Severity Scale (YGTSS) was used to assess the severity of tics, but could only be performed for 127 (48.5%) patients. The control group comprised 279 unrelated, ethnically and gender matched individuals with no diagnosed mental, neurological or general disorders, aged 13–54 years (mean age: 22.5 ± 3.0 years; 200 males, (74.1%). The age of the controls was slightly higher (mean age of the two groups differed only by four years) as we wanted to be sure that the controls have passed the age of tic onset (the onset of GTS after 13 years is very rare).

The patients were evaluated for the clinical diagnosis of GTS and co-morbid mental disorders according to DSM-IV-TR, most of them before the DSM-5 criteria were published. Patients who fulfilled the DSM-5, but not DSM-IV-TR diagnostic criteria for GTS were excluded to ensure homogeneity of group. OCB was diagnosed if obsessions and compulsions were egosyntonic in contrast to egodystonic symptoms which characterized OCD. The diagnosis of co-morbid mental disorders was also made based on earlier psychiatric examinations that had been performed before the time of patients’ evaluation. This included psychiatric disorders that were usually diagnosed in the childhood (e.g., attention deficit hyperactivity disorder or oppositional defiant disorder) and symptoms of which were not yet present in adult patients at the time of examination. All the patients were referred to a neurologist experienced in tic disorders and were personally interviewed by an author of the study (PJ). The study was designed as a one-time registration study and no new clinical data obtained on follow-up visits have been included in the analysis.

### Genetic Analysis and Selection of SNPs

DNA samples were collected between 2007 and 2018. Genomic DNA was extracted either from peripheral blood leukocytes using a standard salting-out procedure (Miller et al., 1988) or from buccal cells collected with Oragene OG-500 DNA collection kit and using Prep IT L2P purification kit (DNA Genotek Inc., Ottawa, Ontario, Canada). The latter method was used with young patients that were unable or unwilling to comply with blood collections. DNA samples obtained with both methods were of same quality and applicability. Genotyping of selected SNPs was performed using TaqMan SNP genotyping assays (Life Technologies, Carlsbad, California, USA) on a StepOne Plus Real-Time PCR system (Life Technologies) (Janik et al., 2014).

*The* rs2023239, rs2180619, rs806379, and rs1049353 *CNR1* polymorphisms were chosen on the basis of their minor allele frequency (MAF) estimated above 15% in both general and Polish populations, and their well-documented association with various neurological and psychiatric disorders (**Table 1**). As indicated by data from the Exome Aggregation Consortium (ExAC) and the Genome Aggregation Database (GnomAD) rs1049353 is the only known polymorphism in the coding sequence of the *CNR1* gene with a MAF above 1%; it is synonymous. All other published variants in the coding sequence are private withfrequency of at most hundreds per 276,000 alleles (MAF below 0.4%) and therefore were not selected for the study. Also in our in-house database of whole exome/genome sequencing results for >200 Polish patients not affected by GTS, the rs1049353 variant is the only one located in the coding sequence of *CNR1*. Thus, except for rs1049353, the selected SNPs are located in non-coding sequences. For clarity we note that rs1049353 is different from the 1359(G/A) polymorphism studied by Gadzicki et al. (Mackie, 2006).

### Statistical Analysis

Chi-square test was used for both allelic and genotypic association studies. Association of genotypes with age of tic onset and YGTSS was analyzed with Kruskal-Wallis test. Multivariate logistic regression model was used to find independent predictors of GTS risk. The study sample size was sufficient to detect with 80% probability the true effect size measured as odds ratio (OR) between 1.62 and 1.97 for positive association or between 0.39 and 0.61 for negative association (depending on the actual MAF which ranged from 0.11 to 0.47) for the differences in allele frequencies of the four polymorphisms between the GTS and the control group. Statistica 13 program was used for statistical calculations. The significance level was set at p < 0.05, and Bonferroni-corrected significance criterion was used where indicated in the text.

## Results

The genotyping success rate was 100% and the consensus rate (on the basis of 10% duplicates) was 100% for DNA isolated from both leukocytes and buccal cells. The quality of genotyping was the same regardless of the biological source and method used for DNA isolation. The genotype frequencies of all four SNPs were in accordance with the Hardy–Weinberg equilibrium in the control group (p>0.9 for rs806379, p>0.3 for rs2023239, p>0.6 for rs2180619 and p>0.6 for rs1049353). In the patient group the genotype frequencies of three SNPs were in accordance with the Hardy–Weinberg equilibrium (p>0.2 for rs2023239, p>0.1 for rs2180619 and p>0.2 for rs1049353) while the distribution of the rs806379 genotype deviated slightly from the Hardy-Weinberg equilibrium (p=0.047) due to a deficit of heterozygotes.

A significant association with the GTS was found for the rs2023239 variant. The MAF of *CNR1* rs2023239 was significantly higher in GTS patients compared to control group. When the TT genotype was used as a reference, genotypes CC+CT (dominant model, p = 0.001) and CT alone (p = 0.0009) were significantly associated with a higher risk of GTS. This association remained significant after Bonferroni correction for multiple tests (Bonferroni-corrected significance level for four SNPs and six genetic models is 0.05/(4*6)=0.002). There were no statistically significant associations between the rs2180619, rs806379 rs1049353 variants and the GTS (**Table 2**). Multivariate logistic regression analysis adjusted for gender and age showed that the presence of *CNR1* rs2023239 allele C (genotypes CC+CT, dominant model) is an independent factor associated with a higher risk of GTS (**Table 3**).

**Table 2:** Genotype and allele distribution of the selected SNPs in patients with Gilles de la Tourette syndrome and controls.

*CNR1* rs2023239						Comparison ^a^	OR ^b^	95%CI		p ^c^
Group	Genotype			Allele		CC+CT vs TT	1.903	1.290	2.808	0.001
	TT	CT	CC	T	C	CC vs CT+TT	1.066	0.305	3.726	0.920
Controls n	222	52	5	496	62	CC vs TT	1.261	0.360	4.426	0.716
%	79.6	18.6	1.8	88.9	11.1	C vs T	1.681	1.188	2.380	0.003
GTS n	176	81	5	433	91	CT vs TT	1.965	1.316	2.933	0.001
%	67.2	30.9	1.9	82.6	17.4	CC vs CT	0.642	0.177	2.327	0.497
*CNR1* rs2180619						Comparison ^a^	OR ^b^	95%CI		p ^c^
Group	Genotype			Allele		GG+AG vs AA	1.281	0.903	1.818	0.164
	AA	AG	GG	A	G	GG vs AG+AA	0.866	0.531	1.412	0.563
Controls n	112	126	41	350	208	GG vs AA	1.032	0.606	1.758	0.909
%	40.1	45.2	14.7	62.7	37.3	G vs A	1.090	0.853	1.393	0.491
GTS n	90	138	34	318	206	AG vs AA	1.363	0.944	1.969	0.099
%	34.4	52.7	13.0	60.7	39.3	GG vs AG	0.757	0.453	1.267	0.289
*CNR1* rs806379						Comparison ^a^	OR ^b^	95%CI		p ^c^
Group	Genotype			Allele		TT+AT vs AA	0.837	0.579	1.210	0.344
	AA	AT	TT	A	T	TT vs AT+AA	1.204	0.807	1.797	0.362
Controls n	78	141	60	297	261	TT vs AA	1.018	0.638	1.625	0.940
%	28.0	50.5	21.5	53.2	46.8	T vs A	0.992	0.781	1.259	0.945
GTS	83	114	65	280	244	AT vs AA	0.760	0.512	1.129	0.173
n										
%	31.7	43.5	24.8	53.4	46.6	TT vs AT	1.340	0.873	2.058	0.181
*CNR1* rs1049353						Comparison ^a^	OR ^b^	95%CI		p ^c^
Group	Genotype			Allele		TT+CT vs CC	0.876	0.622	1.232	0.445
	CC	CT	TT	C	T	TT vs CT+CC	0.626	0.269	1.456	0.273
Controls n	156	108	15	420	138	TT vs CC	0.604	0.257	1.421	0.244
%	55.9	38.7	5.4	75.1	24.9	T vs C	0.865	0.653	1.147	0.314
GTS										
n	155	98	9	408	116	CT vs CC	0.913	0.642	1.299	0.613
%	59.2	37.4	3.4	78.0	22.0	TT vs CT	0.661	0.277	1.579	0.349

^a^Comparison of genotype or allele frequencies between the GTS and control groups, ^b^ OR for the genotype or allele frequencies compared between GTS patients and controls, ^c^ Chi^2^ test

**Table 3:** Multivariate logistic regression analysis of independent factors associated with presence of GTS as dependent variable (GTS patients vs controls).

Independent variables	OR	95%CI		p
Gender (male vs female)	0.922	0.601	1.414	0.710
Age (per 1 year)	0.906	0.879	0.934	<0.00001
*CNR1* rs2023239 (CC+CT vs TT)	1.725	1.146	2.598	0.009

Genotypic and allelic association analysis of all four *CNR1* polymorphisms with most common co-morbid disorders in patients with GTS are shown in **Table 4**. The only significant association was found for rs806379. The minor allele T of this SNP was more frequent in GTS patients with ADHD than in the patients without ADHD (OR 1.45, 95% CI: 1.01–2.08, p=0.045). No associations were found between the *CNR1* genotype and age of tic onset (p>0.4), total YGTSS or YGTSS Total Tic Score (p>0.15), family history of tics (p>0.15) or family history of OCB/OCD (p>0.2).

**Table 4:** Genotypic and allelic association analysis of studied *CNR1* variants with most common co-morbid disorders in patients with Gilles de la Tourette syndrome.

Co-morbid disorder	*CNR1* rs2023239					Comparison ^a^	OR ^b^	95%CI		p ^c^
ADHD	Genotype			Allele		CC+CT vs TT	1.289	0.753	2.206	0.354
	TT	CT	CC	T	C	CC vs CT+TT	0.469	0.052	4.261	0.491
Absent, n	118	49	4	285	57	CC vs TT	0.518	0.057	4.737	0.553
%	69.0	28.7	2.3	83.3	16.7	C vs T	1.164	0.728	1.862	0.525
Present, n	57	32	1	146	34	CT vs TT	1.352	0.783	2.335	0.279
%	63.3	35.6	1.1	81.1	18.9	CC vs CT	0.383	0.041	3.582	0.384
OCD/OCB	Genotype			Allele		CC+CT vs TT	0.619	0.367	1.044	0.071
	TT	CT	CC	T	C	CC vs CT+TT	1.597	0.262	9.717	0.608
Absent, n	83	49	2	215	53	CC vs TT	1.353	0.221	8.299	0.743
%	61.9	36.6	1.5	80.2	19.8	C vs T	0.714	0.452	1.128	0.147
Present, n	92	32	3	216	38	CT vs TT	0.589	0.345	1.006	0.052
%	72.4	25.2	2.4	85.0	15.0	CC vs CT	2.297	0.363	14.518	0.365
Anxiety	Genotype			Allele		CC+CT vs TT	0.947	0.544	1.649	0.848
	TT	CT	CC	T	C	CC vs CT+TT	3.241	0.531	19.772	0.179
Absent, n	118	57	2	293	61	CC vs TT	3.105	0.505	19.106	0.200
%	66.7	32.2	1.1	82.8	17.2	C vs T	1.044	0.645	1.690	0.860
Present, n	57	24	3	138	30	CT vs TT	0.872	0.492	1.545	0.638
%	67.9	28.6	3.6	82.1	17.9	CC vs CT	3.563	0.559	22.695	0.156
Depression	Genotype			Allele		CC+CT vs TT	0.841	0.394	1.793	0.653
	TT	CT	CC	T	C	CC vs CT+TT	1.528	0.166	14.059	0.706
Absent, n	149	71	4	369	79	CC vs TT	1.433	0.154	13.331	0.751
%	66.5	31.7	1.8	82.4	17.6	C vs T	0.904	0.465	1.756	0.766
Present, n	26	10	1	62	12	CT vs TT	0.807	0.369	1.765	0.591
%	70.3	27.0	2.7	83.8	16.2	CC vs CT	1.775	0.180	17.513	0.619
ADHD	Genotype			Allele		GG+AG vs AA	0.864	0.506	1.473	0.590
	AA	AG	GG	A	G	GG vs AG+AA	0.544	0.235	1.257	0.150
Absent, n	57	88	26	202	140	GG vs AA	0.531	0.216	1.308	0.165
%	33.3	51.5	15.2	59.1	40.9	G vs A	0.816	0.562	1.184	0.283
Present, n	33	49	8	115	65	AG vs AA	0.962	0.553	1.672	0.890
%	36.7	54.4	8.9	63.9	36.1	GG vs AG	0.553	0.232	1.314	0.175
OCD/OCB	Genotype			Allele		GG+AG vs AA	0.986	0.592	1.643	0.957
	AA	AG	GG	A	G	GG vs AG+AA	0.531	0.251	1.125	0.095
Absent, n	46	66	22	158	110	GG vs AA	0.570	0.252	1.289	0.175
%	34.3	49.3	16.4	59.0	41.0	G vs A	0.858	0.604	1.220	0.394
Present, n	44	71	12	159	95	AG vs AA	1.125	0.661	1.915	0.665
%	34.7	55.9	9.5	62.6	37.4	GG vs AG	0.507	0.233	1.105	0.084
Anxiety	Genotype			Allele		GG+AG vs AA	0.855	0.497	1.470	0.571
	AA	AG	GG	A	G	GG vs AG+AA	0.730	0.324	1.641	0.444
Absent, n	59	93	25	211	143	GG vs AA	0.685	0.285	1.647	0.397
%	33.3	52.5	14.1	59.%	40.4	G vs A	0.863	0.591	1.260	0.445
Present, n	31	44	9	106	62	AG vs AA	0.900	0.512	1.582	0.715
%	36.9	52.4	10.7	63.%	36.9	GG vs AG	0.761	0.328	1.766	0.524
Depression	Genotype			Allele		GG+AG vs AA	0.844	0.411	1.733	0.643
	AA	AG	GG	A	G	GG vs AG+AA	0.784	0.259	2.370	0.666
Absent, n	76	118	30	270	178	GG vs AA	0.724	0.220	2.376	0.593
%	33.9	52.7	13.4	60.3	39.7	G vs A	0.871	0.523	1.451	0.596
Present, n	14	19	4	47	27	AG vs AA	0.874	0.414	1.847	0.724
%	37.8	51.4	10.8	63.5	36.5	GG vs AG	0.828	0.262	2.616	0.748
Depression	Genotype			Allele		TT+CT vs CC	0.997	0.491	2.024	0.992
	CC	CT	TT	C	T	TT vs CT+CC	0.000	–	–	0.243
Absent, n	133	83	8	349	99	TT vs CC	0.000	–	–	0.252
%	59.4	37.1	3.6	77.9	22.1	T vs C	0.896	0.487	1.648	0.724
Present, n	22	15	0	59	15	CT vs CC	1.093	0.536	2.225	0.807
%	59.5	40.5	0.0	79.7	20.3	TT vs CT	0.000	–	–	0.232
Anxiety	Genotype			Allele		TT+CT vs CC	1.232	0.728	2.085	0.436
	CC	CT	TT	C	T	TT vs CT+CC	1.274	0.297	5.462	0.744
Absent, n	108	64	5	280	74	TT vs CC	1.379	0.316	6.007	0.668
%	61.0	36.2	2.8	79.1	20.9	T vs C	1.182	0.763	1.832	0.453
Present, n	47	34	3	128	40	CT vs CC	1.221	0.712	2.092	0.468
%	56.0	40.5	3.6	76.2	23.8	TT vs CT	1.129	0.254	5.014	0.873
OCD/OCB	Genotype			Allele		TT+CT vs CC	0.796	0.485	1.307	0.367
	CC	CT	TT	C	T	TT vs CT+CC	0.624	0.146	2.667	0.521
Absent, n	76	53	5	205	63	TT vs CC	0.577	0.133	2.499	0.458
%	56.7	39.6	3.7	76.5	23.5	T vs C	0.817	0.539	1.241	0.343
Present, n	79	45	3	203	51	CT vs CC	0.817	0.492	1.356	0.434
%	62.2	35.4	2.4	79.9	20.1	TT vs CT	0.707	0.160	3.121	0.646
ADHD	Genotype			Allele		TT+CT vs CC	1.107	0.659	1.859	0.701
	CC	CT	TT	C	T	TT vs CT+CC	1.942	0.474	7.955	0.348
Absent, n	103	64	4	270	72	TT vs CC	1.981	0.476	8.239	0.339
%	60.2	37.4	2.3	79.0	21.1	T vs C	1.141	0.741	1.759	0.549
Present, n	52	34	4	138	42	CT vs CC	1.052	0.617	1.793	0.851
%	57.8	37.8	4.4	76.7	23.3	TT vs CT	1.882	0.443	8.000	0.385
Anxiety	Genotype			Allele		TT+AT vs AA	1.445	0.811	2.575	0.210
	AA	AT	TT	A	T	TT vs AT+AA	1.106	0.610	2.006	0.741
Absent, n	60	74	43	194	160	TT vs AA	1.395	0.687	2.835	0.356
%	33.9	41.8	24.3	54.8	45.2	T vs A	1.213	0.839	1.752	0.304
Present, n	22	40	22	84	84	AT vs AA	1.474	0.792	2.745	0.220
%	26.2	47.6	26.2	50.0	50.0	TT vs AT	0.947	0.498	1.798	0.867
Depression	Genotype			Allele		TT+AT vs AA	0.716	0.348	1.475	0.364
	AA	AT	TT	A	T	TT vs AT+AA	0.668	0.278	1.603	0.363
Absent, n	68	98	68	234	214	TT vs AA	0.586	0.222	1.550	0.278
%	30.4	43.8	30.4	52.2	47.8	T vs A	0.746	0.452	1.229	0.248
Present, n	14	16	14	44	30	AT vs AA	0.793	0.363	1.732	0.560
%	37.8	43.2	37.8	59.5	40.5	TT vs AT	0.739	0.287	1.903	0.530
OCD/OCB	Genotype			Allele		TT+AT vs AA	0.925	0.548	1.560	0.769
	AA	AT	TT	A	T	TT vs AT+AA	1.031	0.588	1.807	0.915
Absent, n	41	60	33	142	126	TT vs AA	0.970	0.506	1.859	0.926
%	30.6	44.8	24.6	53.0	47.0	T vs A	0.978	0.693	1.379	0.898
Present, n	41	54	32	136	118	AT vs AA	0.900	0.510	1.588	0.716
%	32.3	42.5	25.2	53.5	46.5	TT vs AT	1.077	0.586	1.982	0.810
ADHD	Genotype			Allele		TT+AT vs AA	1.822	1.020	3.255	0.041
	AA	AT	TT	A	T	TT vs AT+AA	1.375	0.771	2.454	0.280
Absent, n	61	71	39	193	149	TT vs AA	1.937	0.960	3.906	0.063
%	35.7	41.5	22.8	56.4	43.6	T vs A	1.448	1.008	2.080	0.045
Present, n	21	43	26	85	95	AT vs AA	1.759	0.943	3.283	0.075
%	23.3	47.8	28.9	47.2	52.8	TT vs AT	1.101	0.590	2.055	0.763

^a^Comparison of genotype or allele frequencies between the GTS patients with and without particular co-morbid disorder, ^b^ OR for the genotype or allele frequencies compared between GTS patients with and without particular co-morbid disorder, ^c^ Chi^2^ test

## Discussion

So far the only comprehensive study investigating genetic variation in the *CNR1* gene in GTS (Janik et al., 2014) was aimed to localize pathogenic mutations in *CNR1* and failed to show differences with healthy controls. Our study is the first to examine common SNP variants of *CNR1* gene in a group of GTS patients. A significant association was found both in the allelic and genotypic analysis between the risk of GTS and the rs2023239 polymorphism. Multivariate analysis proved that CC+CT genotypes are gender-independent factors associated with a higher GTS risk. Our study shows that GTS is associated with rs2023239 polymorphism along with various others neuropsychiatric disorders, such as the cannabis dependence (Benzinou et al., 2008), eating disorders (Yu et al., 2013), schizophrenia (Wiskerke et al., 2012), impulsivity (Juhasz et al., 2009), depression (Ruiz-Contreras et al., 2017), and migraine (Ketcherside et al., 2017). We can only speculate on the molecular mechanism underlying the role of this variant in the pathogenesis of GTS. rs2023239 allele C was found to be associated with increased CB1 receptor density (Janik et al., 2015) which could modulate neurotransmission. It is unlikely that this intronic variant directly affects the splicing, as it is too distant the adjacent exon (122bp). Human Splicing Finder predicts that the minor allele C does not create any new potential splice sites or new potential branch points compared to the common T allele. Additionally, this variant could affect gene expression either directly or through a linkage to functional variant(s) located in regulatory regions.

Our earlier association studies in Polish patients with GTS have revealed a relationship between some SNPs of *BTBD9, ADORA1* and *ADORA2A* genes and co-morbid psychiatric disorders (Muller-Vahl et al., 1999; Martiny, 2017). Therefore, we hypothesized that *CNR1* variants could also contribute to the disease complexity predisposing the patients to psychiatric comorbidities as all the *CNR1* polymorphisms studied here had been found to be associated with various psychiatric disorders (**Table 1**). However, both genotypic and allelic association analyses of all the *CNR1*variants examined, including rs2023239 associated with a higher risk of GTS, failed to demonstrate a significant association with any psychiatric co-morbidity. The only statistically significant association found, that of co-morbid ADHD with rs806379, should probably be regarded as a false positive because of its marginal significance (0.05>p>0.04) in multiple statistical tests performed (4 SNPs x 4 disorders x 6 models).

A number of open as well as randomized controlled studies and case reports support the premise that the use of CBM improves not only tics, but also ADHD (Abi-Jaoude et al., 2017), OCB/OCD (Hasan et al., 2010; Denys et al., 2013), depression (Yoon et al., 2007) and anxiety (Jakubovski and Müller-Vahl, 2017). Our study does not confirm that this beneficial effect of CMB on co-morbid disorders in GTS could be related to *CNR1* SNPs as we did not find any association of the *CNR1* variants with co-morbidity. Additionally, we did not find any correlation of *CNR1* variants with any clinical data, such as age of tic onset, tic severity measured with YGTSS or familial history of tics and OCD/OCB. It can therefore be speculated that while rs2023239 contributes to the pathogenesis of tics *per se*, other genetic and environmental factors contribute to further clinical features of GTS.

The present results on the *CNR1* variants could also be interpreted in relation to an earlier SNP study by our group, in which we found association of *ADORA1* and *ADORA2A* variants with a higher risk of GTS (Muller-Vahl et al., 1999). In this context we note that abnormality of the dopaminergic system has been confirmed to be one of the primary causes of GTS (Singer et al., 1982; Sandyk and Bamford, 1987; Ferré et al., 2009; Ferré et al., 2010; Filbey et al., 2010), both adenosine and endocannabinoids act as modulators of dopamine neurotransmission in the striatum (Müller et al., 2007) and it has been suggested that CB1 receptors form heteromers with dopamine D2 and adenosine A2A receptors (Ehlers et al., 2007). Although we do not know the functional significance of the minor variant of rs2023239 in GTS, we put forward the hypothesis that it could be related to faulty cannabinoid transmission due to lower expression of the CB1 receptor or reduced receptor’s affinity for the endocannabinoid ligand. This, in turn, could lead to reduction of adenosinergic signaling and over-activity of dopaminergic transmission, and finally aggravate tics. Another possible mechanism is a direct enhancement of dopaminergic transmission as a consequence of limited endocannabinoid activity, without an involvement of adenosine signaling.

In conclusion, the obtained results indicate that the C allele of the rs2023239 polymorphism of *CNR1* gene is a risk factor of GTS in the Polish population, associated with the occurrence of tics, but not with the co-existing psychiatric symptoms. The results also support the hypothesis of an ECS involvement in the pathogenesis of GTS. Nevertheless, our finding is preliminary and needs to be replicated in an independent cohort. Further research is needed to determine functional significance of the variant.

## Limitations

The following limitations of the study have to be addressed: 1) the analyzed variants can be in linkage disequilibrium with true risk conferring variants located outside analyzed regions; 2) the study group was relatively small; 3) the patients were evaluated for co-morbid disorders only once (one-time registration) and it cannot be excluded that psychiatric disorders could develop over time and additionally it was impossible to measure the YGTSS in all patients; there is a possibility of false negative results in rs2180619, rs806379, and rs1049353 CNR1 polymorphisms; 4) some clinical data, especially on adult patients, were subject to a recall bias, ; 5) we have not included the comparison with the variants previously associated with GTS and this should be taken into consideration in the future studies; and 6) our finding needs to be replicated using study groups from different populations.

## Data Availability Statement

The raw data supporting the conclusions of this article will be made available by the authors, without undue reservation, to any qualified researcher. These are also included in **Supplementary Material**.

## Ethics Statement

The study has been approved by the Ethics Committee of the Medical University of Warsaw (KB/2/2007, KB/53/A/2010, KB/63/A/2018) and has therefore been performed in accordance with the ethical standards laid down in the 1964 Declaration of Helsinki and its later amendments. All participants aged ≥18 years signed an informed consent form prior to their inclusion in the study, and legal guardians gave a written consent on behalf of individuals under 18.

## Author Contributions

Research project: Conception (PJ), Organization (PJ, CŻ), Execution (JF), Creation of electronic database (TD, NS, PJ), Clinical examination: (PJ), Manuscript Preparation: Writing of the first draft (NS, PJ, JF, KS), Review and Critique (CŻ, PJ, KS, JF, NS, TD).

## Funding

This study was kindly supported by a grant provided by Medical University of Warsaw (1WC/NM1/17) and by a grant funded by National Science Center (UMO-2016/23/B/NZ2/03030).

## Conflict of Interest

The authors declare that the research was conducted in the absence of any commercial or financial relationships that could be construed as a potential conflict of interest.

## Supplementary Material

Supplementary Table 1 The raw data used for analysis in this study.
